# A Systematic Review of Cementation Techniques to Minimize Cement Excess in Cement-Retained Implant Restorations

**DOI:** 10.3390/mps5010009

**Published:** 2022-01-17

**Authors:** Rodolfo Reda, Alessio Zanza, Andrea Cicconetti, Shilpa Bhandi, Renzo Guarnieri, Luca Testarelli, Dario Di Nardo

**Affiliations:** 1 Department of Oral and Maxillo Facial Sciences, University of Rome La Sapienza, 00161 Rome, Italy; rodolforeda17@gmail.com (R.R.); ale.zanza@gmail.com (A.Z.); andrea.cicconetti@uniroma1.it (A.C.); renzoguarnieri@gmail.com (R.G.); dario.dinardo@uniroma1.it (D.D.N.); 2 Department of Restorative Dental Sciences, College of Dentistry, Jazan University, Jazan 45412, Saudi Arabia; shilpa.bhandi@gmail.com

**Keywords:** cementation technique, cement excess, peri-implantitis, shoulder, chamfer

## Abstract

Background: The most used types of retention of implant-supported prostheses are screw-retained or cement-retained restorations. The advantages and disadvantages of both have been identified by various authors over the years. However, cement-retained implant crowns and fixed partial dentures are among the most used types of restorations in implant prostheses, due to their aesthetic and clinical advantages. When cemented prostheses are made on implants, the problem of cement residues is important and often associated with biological implant pathologies. The objective of this research was to establish to what extent the techniques to reduce excess cement really affect the volume of cement residues. Materials and Methods: This review was written following the PRISMA statement; a detailed search was carried out in three different electronic databases—PubMed, Scopus, and Cochrane Library. The inclusion criteria were prospective clinical studies, with at least 10 participants per group, and with at least 6 months of the follow-up period. Results: There have been many proposals for techniques supposed to reduce the amount of excess cement in the peri-implant sulcus and on the prosthetic components, but of these, which are exceptional in their in vitro capabilities, very few have been clinically validated, and this represents the real limitation and a great lack of knowledge regarding this topic. Three articles met the inclusion criteria, which were analyzed and compared, to obtain the information necessary for the purposes of the systematic review. Discussion: Extraoral cementation can reduce the excess cement, which, after a normal excess removal procedure, is, nevertheless, of such size that it does not affect the possibility of peri-implant pathologies developing. All these studies concluded that a small amount of cement residue is found in the gingival sulcus, and using eugenol-free oxide cements, the residues were only deposited on the metal surfaces, with a better peri-implant tissues health. Conclusion: Despite the limitations of this study, it was possible to carefully analyze these characteristics and obtain valuable suggestions for daily clinical practice. Resinous cements are considered, due to the free monomers present in them, toxic for the soft tissues. The provisional zinc-oxide cements, also eugenol-free, represent the ideal choice. The different grades of retentive forces provided by these cements do not seem to have clinical effects on the decementation of restorations.

## 1. Introduction

The most used types of retention of implant-supported prostheses are screw-retained or cement-retained restorations. The advantages and disadvantages of both have been identified by various authors over the years, also following the technological developments that have made the different techniques more and more suitable; nevertheless, cement-retained implant crowns and fixed partial dentures have become a widely used type of restoration in implant prostheses [1,2,3].

The reasons why cement-retained prostheses are the most frequently used restoration are different, but all attributable to aesthetic and clinical advantages. Cement-retained crowns are more aesthetic due to the absence of a screw canal, which allows avoiding aesthetic composite resin reconstructions and allows better-stratified ceramic layers. Moreover, the cement layer between the framework and the implant abutment is presumably an element of prostheses passivation [4,5].

Cement-retained restorations are supposedly more resistant to occlusal load due to a better-stratified ceramic and a higher metal mass [6]. Furthermore, the screw system of abutment retention seems to be less stressed from the various forces that develop in chewing or from the occlusal load in a cement-retained crown than the screw-retained ones [7,8]. One problem in prosthetic restorations on implants is the loosening or fracture of the abutment and retaining screws. Complications such as chronic screw loosening and fracture, in addition to the prosthesis and implant fractures, have been reported [9,10].

All these problems can occur with the two different main types of retention described, with different frequencies depending on the type. In particular, as regards the screw-retained prosthesis, the external hexagon connection was the first solution to be developed and studied. This feature allows easier coupling of the different prosthetic components but worse prosthetic management, with greater gaps and greater presence of the “pump” effect. Although it was simple to use and was considered a valid solution for a long time, higher screws loosening rates and higher marginal bone loss were reported, compared with other solutions. Subsequently, the internal hexagon connection was developed to improve mechanical coupling and system stability [10]. This solution reduced the incidence of microgaps and also demonstrated a better distribution of mechanical stress on the implant surface but, on thin diameter implants, subjects the implant walls at the connection level to high loads, capable of damaging them.

Moreover, cement-retained restorations are the most frequently used restoration in the case of inclined implants with respect to the prosthetic ideal axis, or in the case of non-parallel implants solidarized in the same prosthetic structure [11,12].

The comparison between the screw-retained and the cement-retained prostheses shows that cement-retained ensures a result in terms of bone stability, and therefore implant survival rates [10].

On the other hand, the possibility that cement excess could be not removed from prosthetic restorations or from peri-implant tissue has been thoroughly studied in the literature, particularly in relation to the peri-implant disease. It is certain that the excess of cement can, in some cases, manifest its presence with the typical signs of inflammation at the level of the peri-implant gingival sulcus, giving rise to what is defined as “peri-cementitis” and able to rapidly evolve into the peri-implantitis disease, causing large losses of soft and hard peri-implant tissues [13,14].

Another important topic largely investigated is the supposed better typology of cement used in cement-retained restoration [15,16]. According to Nematollahi F. et al., the two types of cement most frequently used are zinc phosphate and zinc oxide eugenol cements (ZOE); the first shows a simple removal of cement excess due to a weaker adherence to metal and stronger retention, while the second provides easier retrievability but a more difficult cement excess removal [16].

Furthermore, another important risk factor is how deep the implant abutment shoulder is, or the location of the abutment prosthetic finish line. The higher the depth is, the lower the possibility of a complete cement removal [17].

The unremoved cement excess increases the inflammatory condition of the peri-implant tissues due to their components, or by the formation of a favorable substrate for plaque proliferation [14,17].

Due to the deepness of the prosthetic margin location, the abutment angulation, the width of the emergent profile of the crown, and the implant diameter, it is very difficult to completely remove all cement residual [18,19,20,21,22].

Precisely for this reason, it becomes fundamental to reduce the amount of cement during cementing procedures, but it is also important to use an adequate quantity to avoid the decrease in prosthetic rehabilitation retentiveness [17,23].

The purpose of the present systematic review was to evaluate the different cementation techniques present in the literature, but only those that have been clinically validated, in order to understand if some of these could manage to reduce the risk of cement excess residual and its related pathologies.

## 2. Materials and Methods

The review was written following the PRISMA statement for improving the reporting of systematic reviews [24]. A detailed search was carried out in three different electronic databases—PubMed (The National Library of Medicine (MEDLINE, PubMed)), Scopus, and Cochrane Library (The Cochrane Central Register of Controlled Trials (Central)).

The search queries in each database were formulated following the instruction of the PICO model for the clinical question in combination with various Boolean operators. The PICO question asked was “in patients treated with one or more dental implants (P), will cementation techniques to minimize cement excess (I), compared with intraoral cementation technique (C), result in a lower quantity of cement residues and, therefore, in a lower possibility of peri-implant disease cement related development (O)?

The algorithm used, with various Boolean operators, was as follows: ((((cementation) OR (cementing)) AND ((technique) OR (method))) AND ((cement) OR (luting agent))) AND (excess). In addition, a manual search was conducted in the following journals (*The Journal of Prosthetic Dentistry*; *The International Journal of Oral and Maxillofacial Implants*; *Clinical Implant Dentistry*) for possible missing titles (Table 1).

The last search was conducted on 10 January 2021.

The inclusion criteria were prospective clinical studies, with at least 10 participants per group and with at least 6 months of the follow-up period.

The exclusion criteria were in vitro studies, animal studies, experts’ opinions, narrative reviews, editorials, case reports, case series, and articles written in a language other than English. Retrospective studies were also excluded. Only studies exclusively oriented to the study of cement excesses and their biological and microbiological effects were selected. In this way, the research was more homogeneous and between extremely similar studies.

Titles and abstracts were screened by two independent reviewers (R.R., A.Z.). Disagreements were resolved by discussion and with the intervention of a third independent reviewer (L.T.).

Quality assessment was carried out independently by the two reviewers (R.R., A.Z.) as part of the data collection process.

The full texts of all articles of possible interest were obtained, and all relevant data were extracted and entered in a specific form. The level of agreement on study eligibility was evaluated using kappa statistics.

Quality assessment was performed by evaluating articles using the Cochrane risk-of-bias tool, and Newcastle–Ottawa risk scale.

The key domains which were assessed were (1) randomization process, (2) allocation concealment, (3) outcome assessment blinding, (4) data outcome assessment, (5) bias in reporting, and (6) other bias.

Results were assessed by two reviewers (R.R., A.Z.) also in this evaluation, and in case of disagreement, a third reviewer (L.T.) was consulted to achieve consensus.

The judging criteria of the study based on key domains were categorized as a study having a “low” risk of bias when more than four of the key domains were low, and a “high” risk of bias when two or more domains were considered as “high”. If the study did not follow any of the outcomes, it was considered “unclear”.

Only if several studies with overlapping characteristics were found would the meta-analysis be performed.

## 3. Results

With the algorithm used, a total of 1554 articles were found (758 from Scopus, 92 from Cochrane Library, 704 from PubMed). No additional records were found with the manual search from *The Journal of Prosthetic Dentistry*, *The International Journal of Oral and Maxillofacial Implants*, and *Clinical Implant Dentistry*.

After duplicated individuation, 164 articles were excluded. In addition, 1377 articles were excluded after title screening; therefore, 13 articles were assessed for eligibility, and full texts were obtained. From those, only three articles met all the inclusion criteria [17,25,26,27].

The others were excluded because, in their titles, there were no indications about in vitro study, natural teeth study, and retrievability of crown and adhesion strength of cement.

The article “Extraoral Cementation Technique to Minimize Cement-Associated Peri-implant Marginal Bone Loss: Can a Thin Layer of Zinc Oxide Cement Provide Sufficient Retention?” by Frisch E. et al. was excluded because this is a retrospective study, although the title was suitable for inclusion in this study; it focuses on the effects of this cementation technique to decrease excess cement.

There have been many proposals for techniques supposed to reduce the amount of excess cement in the peri-implant sulcus and on prosthetic components, but of these, which are exceptional in their in vitro capabilities, very few have been clinically validated, and this represents a great lack of knowledge regarding this topic.

The three articles selected for this review are from Kiran et al. (2017), from Canullo et al. (2015), and from Frisch et al. (2015).

**Figure 1:**
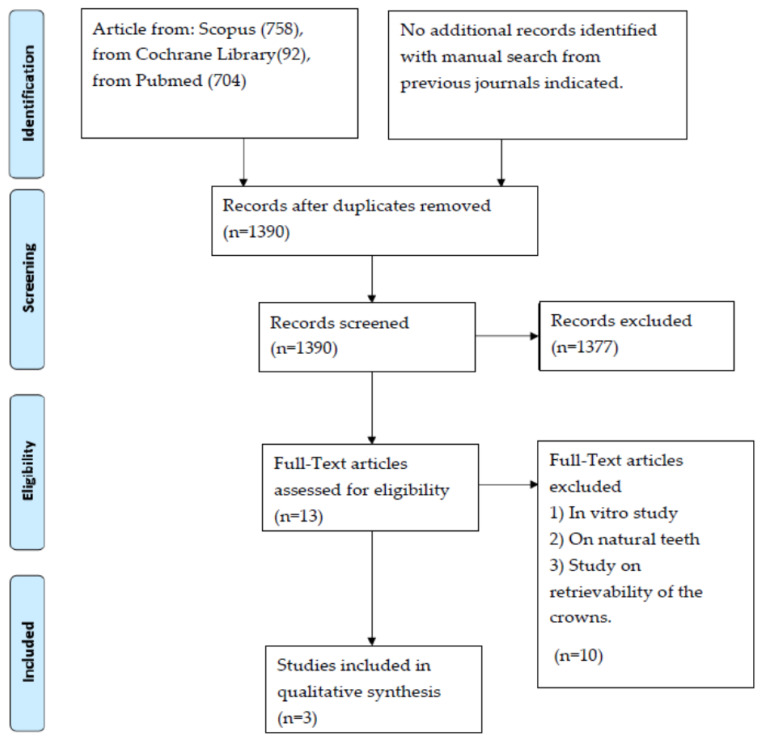
PRISMA search flow results.

PRISMA search flow results are shown in Figure 1. The main characteristics of the three articles selected for this review are indicated in Table 2.

The main characteristic of the two studies necessary to underline is the large number of samples examined—about 90 samples for Canullo et al. and Frisch et al. and only 24 for Kiran et al.; it should be emphasized that the research by Kiran et al. is a split-mouth study, oriented toward the microbiology of oral cavity, which needed to eliminate the variable of different subjects to verify the molecular changes deriving from cementation with two different techniques.

Moreover, the technique used by Kiran et al. to study the excesses of cement and their position was only radiographic (and using radiographs with a parallel cone technique), as well as for Frisch et al. Moreover, Canullo et al. used a much more complex technique to analyze measurements of the surfaces occupied by cement using dedicated software and with magnification.

In the observation period of the various studies, the techniques in which the amount of cement used for intraoral cementation had been decreased, a greater frequency of decementation was reported but never significantly higher.

Regarding the analysis of the quality of the studies, the study by Kiran et al. presents a reduced number of patients, compared with the other two studies considered. However, from the point of view of study planning and execution, following the lines of quality assessment systems used in this research, it is of a high standard. Regarding the technique used to evaluate the amount of excess cement, the one used by Canullo et al. is of a higher level, and this is evident from the higher quality highlighted in this study. The studies are similar by observation period, therefore more easily comparable (Table 3 and Table 4).

In none of the studies, a phenomenon of peri-implantitis related to cement excess was reported.

The results of Cochrane risk of bias for randomized trials and Newcastle–Ottawa risk-of-bias assessment are indicated in Table 3 and Table 4. In the Newcastle–Ottawa risk-of-bias assessment, asterisks indicate the highest grade attributed to the manuscript in that particular characteristic. The follow-up period for each study is indicated in Table 5.

## 4. Discussion

Considering the results obtained, it is evident that further studies need to be carried out to validate the numerous techniques for reducing cement excesses that are constantly being proposed. It is also revealed that more clinical studies are needed to have more reliable information regarding the effectiveness of these techniques and whether they can really improve the therapeutic outcomes by reducing undesired effects [17,18,20].

### 4.1. Cement Excess

All these studies conclude that a small amount of cement residue is found in the gingival sulcus. These conclusions are also highlighted by Behr et al. (2014), who confirmed the almost complete removal of cement residues [17,25,26,27].

In their documented review, Staubli et al. concluded that cemented implant restorations, particularly splinted crowns and fixed partial dentures (FDPs) with submucosal localized crown margins, appear to be at increased risk of excess cement that persists in the submucosal region, subsequently increasing the risk of developing peri-implant diseases. The analysis of the difference between single crowns and small splinted structures will certainly be the subject of future studies, representing today an excellent starting point to exploit a different prosthetic solution [14]. This review, which set similar objectives to this study, shows different results for having mainly analyzed the aspect of the consequence of cement excesses and therefore peri-implant disease.

### 4.2. Type of Cement Used

The use of eugenol-free oxide cement made it possible to find no residues in the soft tissues but only in adhesion to the implant and prosthetic components. It is important to underline that in the study by Kiran et al., the two cementation techniques were used in the same patient, to minimize the differences due to the host’s response to excess cement. Considering that the study evaluated biochemical markers, this foresight was essential [27].

This study confirmed that the health status of periodontal or peri-implant tissues, detected by biomarkers, is very similar. This consideration must always be related to the reduced follow-up of this study, which probably induces an underestimation of the problem [20,27].

Considering the frequency of peri-implantitis and considering the high number of cases using cemented prosthetic rehabilitation, the results of these studies, in particular by Canullo et al., conclude that the possibility of pathologies caused by excess cement is very low [17].

Nevertheless, the choice of the type of cement and the techniques for removing excesses are determining factors and should not be underestimated [17,25].

Unlike the other two studies, the study by Kiran et al. is thoroughly focused on microbiological aspects and inflammation markers, confirming, however, what is demonstrated with clinical measurements of tissue inflammation. Regarding the use of cements, the main division is between definitive and provisional types. Zinc oxide is considered provisional, while glass ionomer, resin, zinc-phosphate, and polycarboxylate cement are considered definitive cements. In the articles considered by this review, mainly zinc oxide was used, and only in the study of Kiran et al., a zinc-polycarboxylate cement was used [17,26,27].

According to Almehmadi’s considerations, the provisional zinc-oxide cements, also eugenol-free, represent the ideal choice to be used as a solution in oral fluids, as they are easily identifiable in intraoral radiography (if with a thickness greater than 1 mm), are easily removed, and allow easier removal of prosthetic restorations, unlike definitive cements [15,28].

These cements provide different grades of retentive force, ranging between 177 N (eugenol-free, zinc-oxide cement) and 813 N (polycarboxylate cement), also underlined by Frisch et al. [26].

However, this different characteristic does not seem to have a clinical effect on the decementation of restorations: It is found from this review that despite the use of different cementation techniques to reduce the formation of excess cement, the decementation of the crowns cemented with temporary cements occurs in a number of cases, certainly greater than conventional techniques but not significant. According to Frisch et al., the rate of premature decementation is 6% after the first 12 months [26].

These data are interesting because they are the results of the longest follow-up study considered by this systematic review, representing sufficient time to assess the presence of peri-implant inflammation. However, there is a greater possibility that crowns cemented on implants with provisional cements and with techniques to reduce cement excess loosen prematurely. According to Schwarz et al., the type of cement (whether permanent or semi-permanent) affects the retrievability of partial dentures but not in single crowns. All of the studies considered in this review, despite obtaining higher values of early decementation, are in line with this statement [29].

With regard to the choice of cement, the importance of its radiopacity must be emphasized. In mesial and distal spaces, it can be extremely important to be able to visualize excess cement on control radiographs, which is further proof that it is possible to use highly reliable temporary cements to cement metal–ceramic crowns on implants, reducing the risks associated with the use of other types of cement. Therefore, this must be a motivation for producing abutments that are more retentive, for example, with secondary retention devices such as coulisses with rougher surfaces, to compensate for the reduced retention forces developed by the provisional cements, as defined by Schiessl et al. in 2013 [30].

### 4.3. Cementation Technique

Kiran et al. affirm that the advantages of the extraoral cementation (EOC) technique can be greater when realized in the aesthetic area, or when the prosthetic margin is very deep. Unlike the other two studies considered, Kiran et al. used locking taper implants, which facilitate the placement of the extraorally cemented abutment crown assembly, not requiring the clinician to cement a crown that has an occlusal hole to allow it to be removed as a whole with the abutment, adding a risk of bias.

It is worth noting, as underlined by Korsch et al., that resinous cements are considered, due to the free monomers present in them, toxic for the soft tissues [31].

Furthermore, Raval et al. demonstrated the ability of eugenol-free zinc-oxide cements to reduce biofilm growth [32].

Despite these results, the presence of these cements in the gingival sulcus should not be underestimated, as they can represent a retention zone for the plaque and offer it the possibility of maturing and stratifying, becoming difficult to remove and particularly dangerous.

Regarding the technique of removing excess cement, this was performed in a very simple way in the studies of Kiran et al. and Frisch et al. mostly with a dental probe in intraoral cementation, and with pellets in extraoral cementation. The excess cement removal procedure was implemented in a more complex way in the study by Canullo et al., in which liquid vaseline, ultrasonic plastic tip, stainless steel explorer, and superfloss were used [17,26,27].

The prosthetic margin was positioned at most 1.5 mm subgingival; even if the indications in this regard are not clear in the literature, this solution has led to very few peri-implant complications [18].

This conclusion, however, finds a further explanation in the rather short follow-up studies on more numerous samples and with 10-year follow-up studies, all of which have revealed high levels of inflammation. Specifically, this assessment must be noted for the results of the study by Kiran et al., which are also justifiable for short follow-ups [33,34,35].

### 4.4. Prosthetic Finish Line

The placement of the prosthetic finish line with respect to the gingival margin represents an extremely important factor, as positioning it more apical than 1.5 mm can represent significant damage and can greatly modify the results and, therefore, the conclusions [21,27].

For the studies considered in this review, exclusively in the study by Canullo et al., the surface characteristics of the abutment and the convergence angle (8° to 12°) were underlined. Further studies are needed to investigate the relationship between these important characteristics [16,29,30].

In the study of Canullo et al., two types of abutments were compared—the first with shoulder and the second with feather edge preparation. Abutments with shoulder preparation were used in all the studies analyzed and have always been the most used solution on implants. In recent years, the feather edge solution, also on implants, is achieving good results and is increasingly widespread, as it guarantees high aesthetic results in a predictable manner [17,36,37,38].

This solution guarantees more space for the soft tissues but generates a greater undercut, due to a higher vertical over-contour, which could make the elimination of cement residues more complex. This study verified that such a possibility does not exist at all and that the excess cement was comparable to those on chamfer shoulder implants, which are appreciated for having a shoulder prosthetic line capable of not causing important over-contours, and, therefore, undercuts [17].

Furthermore, Canullo et al. and Kiran et al. conducted their studies in the posterior areas, where the undercuts are greater, due to the major emergence profile of the crown, and where the removal of cement residues is more complex. Conversely, the study by Frisch et al. has a variable distribution but is still greater in lateral posterior areas [17,26,27].

### 4.5. Cement Residues Visualization

Moreover, the technique of visualization of cement residues, implemented by Canullo et al., is much more complex and specific, certainly more useful for the purposes of the evaluations that were the objective of this systematic review.

The study by Canullo et al. demonstrated that extraoral cementation significantly reduces cement residues at 3 months, compared with a traditional cementation technique [17].

The frequency of early decementation of the implant crowns cemented with the extraoral technique, even if higher, was not significant. Furthermore, it can be concluded that there are no clinically significant differences regarding the two types of abutment designs.

This could indicate that the principles of choosing one solution or the other must not be reduced to this characteristic, as it is almost similar in different methods.

However, chamfer cemented with an intraoral technique presented a significantly higher amount of cement remnants, compared with a shoulderless abutment when extraoral cementation technique is adopted, but not as high, compared with the chamfer when an extraoral technique is adopted [17].

Despite the limitations of this study, it was possible to extrapolate different suggestions from the three articles that can guide the clinician toward a more correct use of some techniques. These should be evaluated as suggestions and never as definitive conclusions; although a systematic review, this article presents a small number of articles that met the inclusion criteria. The noteworthy contribution of this systematic review is that it represents the starting point for a series of studies, especially in vivo, which is considerably lacking in the literature, to answer the main questions about these different techniques.

## 5. Conclusions

The main limitation of this study is the identification of only three articles that met the inclusion criteria, and therefore, the conclusions must be considered rather as general indications that were possible to obtain, and which we hope will guide toward a greater number of publications on this topic.

From these articles, it is possible to highlight how extraoral cementation can reduce the excess cement, which, after a normal excess removal procedure, is, nevertheless, of such size that it would not affect the possibility of peri-implant pathologies developing.

It is, therefore, possible to define the effectiveness of this technique, despite the presence of subgingival margins (1,5 mm); the presence of peri-implant disease was nil after 1 year of observation, which can be interpreted as the total absence of excess cement in the gingival sulcus. Furthermore, the low percentages of decementation suggest that it is possible to use this technique, in daily clinical practice.

The following conclusions were drawn from this review:

- All the three studies evaluated in this review conclude that a small amount of cement residue is found in the gingival sulcus;
- The use of eugenol-free oxide cement made it possible to find no residues in the soft tissues but only in adhesion to the implant and prosthetic components;
- Pathologies caused by excess cement are low;
- Resinous cements should be considered, due to the free monomers present in them, toxic for the soft tissues;
- The provisional zinc-oxide cements, also eugenol-free, seem to represent the ideal choice to be brought into solution in oral fluids, as they are easily recognizable in intraoral radiography, are easily removed, and allow easier removal of prosthetic restorations, unlike definitive cements;
- The different grades of retentive forces provided by these cements do not seem to have a clinical effect on the decementation of the restorations.

## Figures and Tables

**Table 1:** Search algorithm used.

Search Algorithm	Database	Results
((((cementation) OR (cementing)) AND ((technique) OR (method))) AND ((cement) OR (luting agent))) AND (excess).	Scopus	758
((((cementation) OR (cementing)) AND ((technique) OR (method))) AND ((cement) OR (luting agent))) AND (excess).	Cochrane Library	92
((((cementation) OR (cementing)) AND ((technique) OR (method))) AND ((cement) OR (luting agent))) AND (excess).	Pubmed	704

**Table 2:** Main characteristics of the articles included in the study.

Studies	Year	Sample Size	Sample Characteristics (and Follow-Up)	Implant Type	Implant Subgingival Location	Type of Prostheses	Type of Cementation	Type of Cement	Type of Cement Remotion	Cement Remnants Analysis
Kiran et al.	2017	12 patients 24 implants	Bilateral-single tooth gap (extraction no later than 6 months before), presence of M-D teeth, -------- Follow up 6 months	Locking taper connection (this allows the crown to be extraorally cemented without drilling it to connect it to the implant)	1 mm subgingival (abutment with shoulder preparation)	Metal ceramic crown	Extraoral (abutment with locking taper connection) - Intraoral	Zinc Polycarboxylate	Not Described	X-ray periapical radiograph long-cone paralleling technique
Canullo et al.	2015	46 patients 90 implants	2 adjacent implants posterior maxilla ----------- Follow up 3 months	Screwed Abutment 8–12° of convergence	1.5 mm subgingival (abutment with shoulder or with feath er edge)	Metal ceramic crown (with occlusal opening closed with composite resin)	Extraoral Intraoral Thin layer of Vaseline around the external margin, cement positioned with micro brush, polyurethane resin replica	Eugenol free Zinc Oxide cement	Stainless steel explorer + ultrasonic plastic tip + superfloss (using magnifying loops)	Optical light microscope analysis of the occupied surface by cement remnants
Frisch et al.	2015	68 patients 92 implants	Variable anatomical distribution, single crown Surgery performed in the same center. ---- Follow-up 12 months	Screwed abutment	1.5 mm subgingival (abutment with shoulder preparation)	Metal ceramic crown	Extraoral Intraoral Resin replica of the internal crown side	Zinc oxide cement	Extraoral From Replica with synthetic pellets. Intraoral Dental probe only.	X-ray periapical radiographs Long-cone paralleling technique

**Table 3:** Risk of bias of the included studies according to Cochrane risk of bias for randomized, controlled trials.

Studies	Random Sequence Generation	Allocation Concealment	Blinding of Participants and Personnel	Blinding of Outcome Assessment	Incomplete Outcome Data	Selective Reporting
Kiran et al. 2017	Low	Low	High	High	Low	Low
Canullo et al. 2015	Low	Low	High	High	Low	Low
Frisch et al. 2015	Unclear	High	High	High	Low	High

**Table 4:** Newcastle–Ottawa risk-of-bias assessment.

Study	Selection (***)			Comparability (**)	Outcome (**)		Total
	Representativeness of exposed Cohort (*)	Selection of non exposed Cohort (*)	Ascertainment of exposure (*)		Assessment of outcome (*)	Adequacy of follow-up (*)	
Kiran et al. 2017	*			*	*		3
Canullo et al. 2015	*	*	*	**	*		6
Frisch et al. 2015	*	*		*	*	*	5

**Table 5:** Follow-up period for each study.

Study	Type of the Study	Follow-Up
Kiran et al. 2017	Prospective clinical study	6 months
Canullo et al. 2015	Randomized controlled prospective clinical study	3 months
Frisch et al. 2015	Prospective clinical study	12 months

## References

[1] Wittneben J.G., Joda T., Weber H.P., Brägger U. (2017). Screw retained vs. cement retained implant-supported fixed dental prosthesis. Periodontology.

[2] Wittneben J.G., Millen C., Brägger U. (2014). Clinical Performance of Screw-Versus Cement-Retained Fixed Implant-Supported Reconstructions-A Systematic Review. Int. J. Oral Maxillofac. Implant..

[3] Alraheam I.A., Ngoc C.N., Wiesen C.A., Dds T.E.D. (2018). Five-year success rate of resin-bonded fixed partial dentures: A systematic review. J. Esthet. Restor. Dent..

[4] Misch C.E. (1995). Screw-retained versus cement-retained implant-supported prostheses. Pract. Periodont. Aesthet. Dent..

[5] Chee W.W., Duncan J., Afshar M., Moshaverinia A. (2013). Evaluation of the amount of excess cement around the margins of cement-retained dental implant restorations: The effect of the cement application method. J. Prosthet. Dent..

[6] Tribst J.P.M., Piva A.M.D.O.D., Penteado M.M., Borges A.L.S., Bottino M.A. (2018). Influence of ceramic material, thickness of restoration and cement layer on stress distribution of occlusal veneers. Braz. Oral Res..

[7] Tabata L.F., Assunção W.G., Barão V.A.R., de Sousa E.A.C., Gomes É.A., Delben J.A. (2010). Implant platform switching: Biomechanical approach using two-dimensional finite element analysis. J. Craniofacial Surg..

[8] Passaretti A., Petroni G., Miracolo G., Savoia V., Perpetuini A., Cicconetti A. (2018). Metal free, full arch, fixed prosthesis for edentulous mandible rehabilitation on four implants. J. Prosthodont. Res..

[9] Sinjari B., D’Addazio G., Traini T., Varvara G., Scarano A., Murmura G., Caputi S. (2019). A 10-year retrospective comparative human study on screw-retained versus cemented dental implant abutments. J. Biol. Regul. Homeost. Agents.

[10] Scarano A., Murmura G., Sinjiari B., Sollazzo V., Spinelli G., Carinci F. (2011). Analysis and Structural Examination of Screw Loosening in Oral Implants. Int. J. Immunopathol. Pharmacol..

[11] Camatta H.P., Ferreira R.M., Ferrairo B.M., Strelhow S.S., Rubo J.H., Mori A.A., Ferruzzi F. (2020). Mechanical Behavior and Fracture Loads of Screw-Retained and Cement-Retained Lithium Disilicate Implant-Supported Crowns. J. Prosthodont..

[12] Apaza Alccayhuaman K.A., Soto-Peñaloza D., Nakajima Y., Papageorgiou S.N., Botticelli D., Lang N.P. (2018). Biological and technical complications of tilted implants in comparison with straight implants supporting fixed dental prostheses. A systematic review and meta-analysis. Clin. Oral Implant. Res..

[13] Pesce P., Canullo L., Grusovin M.G., De Bruyn H., Cosyn J., Pera P. (2015). Systematic review of some prosthetic risk factors for periimplantitis. J. Prosthet. Dent..

[14] Staubli N., Walter C., Schmidt J.C., Weiger R., Zitzmann N.U. (2016). Excess cement and the risk of peri-implant disease—A systematic review. Clin. Oral Implant. Res..

[15] Almehmadi N., Kutkut A., Al-Sabbagh M. (2019). What is the Best Available Luting Agent for Implant Prosthesis?. Dent. Clin..

[16] Nematollahi F., Beyabanaki E., Alikhasi M. (2015). Cement Selection for Cement-Retained Implant-Supported Prostheses: A Literature Review. J. Prosthodont..

[17] Canullo L., Cocchetto R., Marinotti F., Penarrocha-Oltra D., Diago M.P., Loi I. (2015). Clinical evaluation of an improved cementation technique for implant-supported restorations: A randomized controlled trial. Clin. Oral Implant. Res..

[18] Linkevicius T., Puisys A., Vindasiute E., Linkeviciene L., Apse P. (2012). Does residual cement around implant-supported restorations cause peri-implant disease? A retrospective case analysis. Clin. Oral Implant. Res..

[19] Linkevicius T., Puisys A., Steigmann M., Vindasiute E., Linkeviciene L. (2014). Influence of Vertical Soft Tissue Thickness on Crestal Bone Changes Around Implants with Platform Switching: A Comparative Clinical Study. Clin. Implant Dent. Relat. Res..

[20] Vindasiute E., Puisys A., Maslova N., Linkeviciene L., Peciuliene V., Linkevicius T. (2013). Clinical Factors Influencing Removal of the Cement Excess in Implant-Supported Restorations. Clin. Implant Dent. Relat. Res..

[21] Linkevicius T., Vindasiute E., Puisys A., Peciuliene V. (2011). The influence of margin location on the amount of undetected cement excess after delivery of cement-retained implant restorations. Clin. Oral Implant. Res..

[22] Sancho-Puchades M., Crameri D., Özcan M., Sailer I., Jung R.E., Hämmerle C.H.F., Thoma D.S. (2017). The influence of the emergence profile on the amount of undetected cement excess after delivery of cement-retained implant reconstructions. Clin. Oral Implant. Res..

[23] Frisch E., Ratka-Kruger P., Weigl P., Woelber J. (2016). Extraoral cementation technique to minimize cement-associated Peri-implant marginal bone loss: Can a thin layer of zinc oxide cement provide sufficient retention. Int. J. Prosthodont..

[24] Moher D., Liberati A., Tetzlaff J., Altman D.G., Prisma Group (2009). Preferred reporting items for systematic reviews and meta-analyses: The PRISMA statement. PLoS Med..

[25] Behr M., Spitzer A., Preis V., Weng D., Gosau M., Rosentritt M. (2014). The extent of luting agent remnants on titanium and zirconia abutment analogs after scaling. Int. J. Oral Maxillofac. Implant..

[26] Frisch E., Ratka-Krüger P., Weigl P., Woelber J. (2015). Minimizing excess cement in implant-supported fixed restorations using an extraoral replica technique: A prospective 1-year study. Int. J. Oral Maxillofac. Implant..

[27] Kıran B., Toman M., Buduneli N., Lappin D.F., Toksavul S., Nizam N. (2017). Intraoral versus extraoral cementation of implant-supported single crowns: Clinical, biomarker, and microbiological comparisons. Clin. Implant Dent. Relat. Res..

[28] Wadhwani C., Piñeyro A., Hess T., Zhang H., Chung K.-H. (2011). Effect of implant abutment modification on the extrusion of excess cement at the crown-abutment margin for cement-retained implant restorations. Int. J. Oral Maxillofac. Implant..

[29] Schwarz S., Schröder C., Corcodel N., Hassel A.J., Rammelsberg P. (2011). Retrospective Comparison of Semipermanent and Permanent Cementation of Implant-Supported Single Crowns and FDPs with Regard to the Incidence of Survival and Complications. Clin. Implant Dent. Relat. Res..

[30] Schiessl C., Schaefer L., Winter C., Fuerst J., Rosentritt M., Zeman F., Behr M. (2012). Factors determining the retentiveness of luting agents used with metal- and ceramic-based implant components. Clin. Oral Investig..

[31] Korsch M., Walther W., Marten S.M., Obst U. (2014). Microbial analysis of biofilms on cement surfaces: An investigation in cement-associated peri-implantitis. J. Appl. Biomater. Funct. Mater..

[32] Raval N.C., Wadhwani C.P., Jain S., Darveau R.P. (2015). The interaction of implant luting cements and oral bacteria linked to peri-implant disease: An in vitro analysis of planktonic and biofilm growth–a preliminary study. Clin. Implant Dent. Relat. Res..

[33] Guarnieri R., Miccoli G., Reda R., Mazzoni A., Di Nardo D., Testarelli L. (2021). Laser microgrooved vs. machined healing abutment disconnection/reconnection: A comparative clinical, radiographical and biochemical study with split-mouth design. Int. J. Implant Dent..

[34] Guarnieri R., Di Nardo D., Di Giorgio G., Miccoli G., Testarelli L. (2021). Evaluation of peri-implant tissues condition after 10–15 years of loading in treated chronic periodontitis patients attending a private practice setting: A retrospective study. Clin. Oral Implant. Res..

[35] Guarnieri R., Miccoli G., Reda R., Mazzoni A., Di Nardo D., Testarelli L. (2021). Sulcus fluid volume, IL-6, and Il-1b concentrations in periodontal and peri-implant tissues comparing machined and laser-microtextured collar/abutment surfaces during 12 weeks of healing: A split-mouth RCT. Clin. Oral Implant. Res..

[36] Cocchetto R., Canullo L. (2015). The “hybrid abutment”: A new design for implant cemented restorations in the esthetic zones. Int. J. Esthet. Dent..

[37] Scutellà F., Weinstein T., Lazzara R., Testori T. (2015). Buccolingual implant position and vertical abutment finish line geometry: Two strictly related factors that may influence the implant esthetic outcome. Implant Dent..

[38] Kurt A., Altintas S.H., Kiziltas M.V., Tekkeli S.E., Guler E.M., Kocyigit A., Usumez A. (2018). Evaluation of residual monomer release and toxicity of self-adhesive resin cements. Dent. Mater. J..

